# Epileptic Mechanisms Shared by Alzheimer’s Disease: Viewed via the Unique Lens of Genetic Epilepsy

**DOI:** 10.3390/ijms22137133

**Published:** 2021-07-01

**Authors:** Jing-Qiong Kang

**Affiliations:** 1 Department of Neurology & Pharmacology, Vanderbilt University Medical Center, Nashville, TN 37232-8552, USA; jingqiong.kang@vumc.org; Tel.: +1-615-936-8399; Fax: +1-615-322-5517; 2 Vanderbilt Kennedy Center of Human Development, Vanderbilt Brain Institute, Vanderbilt University, Nashville, TN 37232-8522, USA

**Keywords:** genetic epilepsy (GE), Alzheimer’s disease (AD), amyloid β (Aβ), electroencephlography (EEG), excitatory/inhibitory(E/I) imbalance, gamma-aminobutyric acid (GABA), GABAergic signaling, endoplasmic reticulum (ER) stress, protein trafficking

## Abstract

Our recent work on genetic epilepsy (GE) has identified common mechanisms between GE and neurodegenerative diseases including Alzheimer’s disease (AD). Although both disorders are seemingly unrelated and occur at opposite ends of the age spectrum, it is likely there are shared mechanisms and studies on GE could provide unique insights into AD pathogenesis. Neurodegenerative diseases are typically late-onset disorders, but the underlying pathology may have already occurred long before the clinical symptoms emerge. Pathophysiology in the early phase of these diseases is understudied but critical for developing mechanism-based treatment. In AD, increased seizure susceptibility and silent epileptiform activity due to disrupted excitatory/inhibitory (E/I) balance has been identified much earlier than cognition deficit. Increased epileptiform activity is likely a main pathology in the early phase that directly contributes to impaired cognition. It is an enormous challenge to model the early phase of pathology with conventional AD mouse models due to the chronic disease course, let alone the complex interplay between subclinical nonconvulsive epileptiform activity, AD pathology, and cognition deficit. We have extensively studied GE, especially with gene mutations that affect the GABA pathway such as mutations in GABA_A_ receptors and GABA transporter 1. We believe that some mouse models developed for studying GE and insights gained from GE could provide unique opportunity to understand AD. These include the pathology in early phase of AD, endoplasmic reticulum (ER) stress, and E/I imbalance as well as the contribution to cognitive deficit. In this review, we will focus on the overlapping mechanisms between GE and AD, the insights from mutations affecting GABA_A_ receptors, and GABA transporter 1. We will detail mechanisms of E/I imbalance and the toxic epileptiform generation in AD, and the complex interplay between ER stress, impaired membrane protein trafficking, and synaptic physiology in both GE and AD.

## 1. Introduction

Alzheimer’s disease (AD) is a common form of dementia and a massive public health burden, but there is currently no effective treatment that can modify the disease process or delay the disease onset. It is estimated that AD affects up to one third of the population over age 85 [1], and it has become an even more urgent health care burden worldwide given the increased life expectancy of the aging population. Although genetic mutations such as amyloid precursor protein (APP), PSEN1, or PSEN2 have been identified in AD [2,3,4], the clinical symptoms of AD, including progressive dementia, occur sporadically in the majority of cases, with no clear genetic risk factor. The treatment for AD focused on the clearance of amyloid β has failed in multiple clinical trials. This thus calls for a broader view of AD pathophysiology with further elucidation of disease mechanisms and identification of better therapeutic targets.

## 2. Major Established Pathological Features of AD

AD as a disease entity has been known for over 100 years. The pathological hallmarks of AD are extracellular amyloid plaques and intraneuronal neurofibrillary tangles, whose building blocks are amyloid-β (Aβ) peptides and phosphorylated tau, respectively. Aβ peptides are generated from APP encoded by the *APP* gene. The cerebral accumulation of Aβ oligomers is believed to be the initial step in the disease process [5]. Aβ is a proteolytic fragment of transmembrane APP after cleavages by β- and γ-secretases. Mutations in genes encoding APP as well as β- and γ-secretases have been identified in human. Animal models carrying the same gene mutations resembled the pathology identified in AD patients and revealed a loss of function mechanism of the mutant gene in familial AD [6,7], reinforcing the causative role of APP, PSEN1, and PSEN2 in AD pathogenesis. 

AD pathophysiology is multifaceted and involves enormous changes of signaling pathways at the molecular level. It is generally accepted that the histopathological findings in AD can be categorized into four central mechanisms: amyloid plaques (Aβ accumulation), neurofibrillary tangles tau hyperphosphorylation, neuroinflammation, and vascular dysfunction. Tau is a brain-specific, axon-enriched, microtubule-associated protein and can be phosphorylated by an array of kinases. The major biological function of tau protein is to stabilize microtubules. Tau proteins are abundant in neurons but are expressed at very low levels in the central nervous system (CNS) in astrocytes and oligodendrocytes. In the AD brain, tau is hyperphosphorylated [8]. Tau hyperphosphorylation is also observed in traumatic brain injury (TBI) [9], an established risk factor for epilepsy, as well as for cognitive dysfunction. This suggests the existence of common underlying molecular underpinnings for AD and epilepsy. In addition to Aβ deposition and tau pathology, other major pathological features, such as neuroinflammation and vascular dysfunction, also contribute to and are reciprocally affected by the formation of Aβ plaques and tangles in AD development and progression [10,11]. The macroscopic changes of AD brains are characterized by parenchymal atrophy and enlarged ventricles, due to progressive neuronal loss beginning in and spreading from the entorhinal cortex in the medial temporal lobe [12,13]. These gross changes in relevant brain regions for memory and executive function correlate with impaired cognition in AD [14].

## 3. Increased Epileptiform Activity or Seizures in AD: An Emergent Common but Under-Recognized Feature

Recent studies from our own group and others suggest that subclinical or nonconvulsive epileptiform activity or seizures are an emergent common but under-recognized feature of AD [15,16,17,18]. On the other hand, several epilepsy models have shown significant correlation between seizure occurrence, neuronal death, and cognitive dysfunction. This suggests a complex interplay between seizure activity and cognitive dysfunction [19]. The increased abnormal EEG activity may exist long before the impaired cognition emerges at a clinical level. It has been reported that subclinical epileptiform activity was detected in over 40% of AD patients, which is four times higher than in controls [16]. It is very possible that these toxic brain electrical discharges may themselves be an important driver of cognitive decline [16,20,21,22,23,24], independently or as part of an interaction with Aβ pathobiology (phosphorylated tau, oxidative damage, neurotransmitter imbalance, and neuronal/synapse loss). These subclinical epileptiform changes are more common than previously believed but often go unnoticed because of their subtle clinical effects, particularly because epileptiform spikes were detected primarily during sleep in AD [25,26]. Importantly, unprovoked seizures and subclinical epileptiform activity were reported at the prodromal stages of AD, starting in some cases long before both clinical symptoms and alterations in brain imaging, suggesting a role for this process in the early pathogenesis of AD [27].

Patients with childhood onset epilepsy show accelerated aging, including a greater Aβ burden 5 decades after diagnosis compared to nonepileptic controls [28]. Although the etiology of absence epilepsy in the studied cohort is largely unknown, some may have the APOEϵ4 allele, which can predispose the patients to the development of progressive neurodegenerative disorders, such as AD. This suggests a bidirectional link between AD and epilepsy. Abnormal neuronal activity, such as brain hyperexcitability, may contribute to susceptibility to AD neuropathology independent of the Aβ metabolic pathway and cholinergic system and may also result directly from early stages of Aβ accumulation. Increased abnormal brain hyperexcitability and seizures have been observed in several mouse models of AD [21], including data from our own group showing the direct effects of even mild seizures on cognition in young *APP/PSEN1* mice [29]. Due to the conservation of intracellular protein quality control mechanisms [30,31] and shared molecular pathophysiology among diseases with misfolded/aggregated proteins, mice that were developed to study epilepsy and developed mutant protein aggregation could be repurposed to study AD and neurodegeneration. These may provide unique novel insights that cannot be obtained from conventional AD animal models. The prevention or suppression of toxic subclinical epileptiform activity is therefore a useful approach to improve cognition in AD, regardless of disease etiology at the molecular and cellular level, such as Aβ or tau pathology.

## 4. Increased EEG Abnormalities in AD Patients

It is well known that patients suffering from certain types of cognitive disorders, including AD, frontotemporal dementia, and Lewy body dementia, have a high risk of developing seizures [24,27]. In cognitively asymptomatic mutation carriers for autosomal dominant AD, there is an increased number of patients who have experienced seizures [32,33]. On the other hand, patients with recurrent seizures suffer cognitive decline and have a higher risk for cognitive impairment. This correlation of severe recurrent seizures and cognitive dysfunction has been established for both genetic and acquired epilepsies [34]. It has been hypothesized that epileptic seizures and postictal confusion may have an important negative effect on the cognitive performance of AD patients. A recent study with EEG recordings in AD patients suggests that the increase in relative theta power may be the first change in patients before dementia [35]. This finding leads to a conclusion that quantitative EEG (qEEG) power could potentially be used as a diagnostic tool for AD, in which some EEG signatures can serve as a biomarker in AD. This echoes the fact that the first description of Aβ came from the neurological examination of an epilepsy patient by Blocq and Marinesco more than a century ago and suggests a strong link between epilepsy and AD [36].

A prominent feature of AD is increased hippocampal excitability. Interestingly, in temporal lobe epilepsy, Aβ plaques are significantly higher, suggesting a common neural mechanism underlying both diseases. In both AD patients and animal models of AD, a chronic aberrant increase of excitatory neuronal activity was detected [37]. Recent studies on several AD animal models, including work from our own group, show increased neuronal network excitability, epileptiform EEG activity, and nonconvulsive seizures, and an increase in the proportion of abnormally hyperactive neurons in cortical circuits and a single seizure event could exacerbate cognitive dysfunction [38]. On the other hand, some studies suggest that hyperexcitation in neural circuitry also promotes APP deposition and spike bursts. Spike bursts, in return, induce the conformational change of the presenilin 1 (PS1) subunit of γ-secretase and increase the Aβ 40/42 ratio [39]. This suggests a complex bidirectional interplay between Aβ deposition and hyperexcitation in neural circuitry. Furthermore, studies indicate that the occurrence of seizures was associated with higher mortality rates in animal models of AD [29]. As to seizure types, generalized convulsive seizures are rare in the whole AD population [40], although they are relatively common in genetic forms of AD, such as patients with *APP* or *PSEN1* mutations. It is likely that nonconvulsive subclinical seizures are more frequent in AD. These subclinical events often go unnoticed or are considered signs of episodic confusion in AD patients, thus obscuring the true picture of seizure activity in AD [41,42].

There exists a complex interplay between AD pathology and brain hyperexcitability. We identified that in an App/Psen1 mouse model, a single seizure episode could irreversibly impair cognition [29]. This reinforces the hypothesis that increased seizure activity itself contributes to impaired cognition. Along the same line, antiepileptic treatment had a positive effect on cognitive function in both animal and human studies. As the detection of seizures in patients with cognitive decline is extremely difficult due to methodological problems, the true prevalence of seizures has remained unclear. Nevertheless, the link between seizures either at the electronic or clinical level and cognitive dysfunction has been established.

## 5. Excitatory/Inhibitory (E/I) Imbalance Contributes to Altered Hyperexcitability and Cognitive Decline in AD

A delicate E/I balance or synaptic homeostasis is essential for normal brain circuit function, and subtle disturbances of this delicate balance can lead to pathological conditions, including seizures and cognition deficits [43,44,45]. Changes at multiple molecular and cellular components, such as receptors, transporters, scaffold proteins, etc., can all contribute to E/I balance. In the pediatric population, disrupted E/I balance has been proposed to be the pathophysiology for epilepsy [46] and other common comorbidities, such as autism [47]. Although there are non-ion channel genes identified, GE is often associated with mutations in gene-encoding ion channels or transporters, suggesting the contribution of altered ion flow and membrane potential. In AD, the accumulation of Aβ peptides in the brain is an essential feature. This suggests that the disease process may start long before the onset of overt cognitive symptoms and EEG abnormality. It is likely that during this very extended early phase of disease pathology, soluble Aβ oligomers and amyloid plaques chronically remodel the function of brain cells, including neurons, astrocytes, and microglia, at the cellular level by the very presence of Aβ oligomers and amyloid plaques. The changed cell function can consequently modify neuronal circuitry and function. It is possible that the alteration can occur at local neuronal circuits or that large-scale networks are altered, leading to a disrupted synaptic E/I balance in the brain [38].

It has been established that Aβ can disrupt the E/I balance, causing hyperactivity in cortical and hippocampal neurons. This consequently results in a breakdown of slow-wave oscillations and network hypersynchrony. It is worth noting that the hyperactivity of hippocampal neurons precedes amyloid plaque formation. This suggests that neuronal hyperactivity is one of the earliest dysfunctions in the pathophysiological cascade initiated by abnormal Aβ accumulation. Therapeutic approaches that correct the E/I balance in the early phase of AD may prevent neuronal dysfunction, neuronal degeneration, and cognitive impairments that emerge in later phases of AD. It has been established that excitatory synaptic transmission is driven mainly by glutamatergic synapses, whereas inhibitory synaptic transmission is mainly mediated by GABAergic and glycinergic signaling. In addition to phasic synaptic neuroinhibition, tonic ion conductance plays an important role in controlling overall neuronal excitability [48]. Enhanced tonic inhibition has been observed in multiple models of absence seizures [49]. This notion is reinforced by the recent association of mutations in the GABA transporter encoding *SLC6A1* and epilepsy and impaired cognition, for which partial or complete loss of the GABA transporter 1 function has been identified as common mechanisms [50,51,52,53]. Together, these systems provide the molecular underpinnings for abnormal EEG, impaired cognition in the brain, and how disruption in these systems would compromise brain function.

The disruption of both GABAergic and glutamatergic systems is accepted as an important modifier of AD [54,55,56,57], but the specific contributions of each have not been fully elucidated. The discovery that GABAergic interneurons are targets of the Aβ peptide suggests that deregulation of the E/I balance contributes to changes in cortical regulation, possibly with consequences for the development of the pathology. Thus, understanding the molecular details involved in GABAergic alterations, in addition to previously recognized glutamatergic neurotransmission, may provide insight into the pathogenesis of AD [58].

## 6. The Overlapping Mechanisms Identified in GE Associated with Mutations in *GABRG2* and AD

Genetic advances have identified many mutations in genes encoding ion channels, transporters, kinases, and transcriptional factors [50,59] that can give rise to a variety of epilepsy syndromes in the pediatric population. Many of these mutations can cause refractory epilepsy and impaired cognition, which collectively are called epileptic encephalopathy. It is intriguing that mutations in the same gene can give rise to mild or severe epileptic encephalopathy phenotypes. We have extensively studied the molecular pathophysiology of the mutations in *GABRG2* associated with either mild or severe epilepsy in vitro to in vivo. We demonstrated that the mutations in *GABRG2* associated with severe epilepsy have mutant protein accumulation and aggregation, while the mutations in the same gene associated with mild epilepsy do not have the mutant protein accumulation and aggregation [30,60]. Surprisingly, the mutant protein aggregates are only detected in old mice (around 1 year old) but not in young mice (under 4 months old). However, the signal of the mutant protein in the neuronal somata region is increased in the young mice and is further increased over time. This suggests that the mutant protein accumulation and formation of protein aggregates are chronic and ongoing processes. In addition to GABA_A_ receptors, ER retention and associated degradation are also major mechanisms for GABA transporter 1 encoding *SLC6A1* gene mutations as demonstrated in our recent studies [51]. The mice with the *GABRG2* mutation that produce protein aggregates at old age had more frequent and prolonged ictal discharges compared with the mice with the loss of the same gene allele function but without mutant protein production [60]. In AD, seizures can be manifested as an early symptom [32]. Similarly, we have identified EEG abnormality and seizure activity in the AD mouse model App/Psen1 [29]. In addition to AD, abnormal EEG has been identified in other neurodegenerative diseases, such as Parkinson’s and Huntington’s diseases, suggesting a correlation between neuronal dysfunction and neuronal cellular injury.

## 7. Insights from GE into Understanding E/I Imbalance in AD

Epilepsy is widely viewed as a disorder of neuronal and network excitability. The pathophysiological hallmarks of seizure generation are hyperexcitability of individual neurons and hypersynchronous firing of neuronal networks. This implies, in a given neuron, the level of membrane depolarization that must be exceeded to a threshold that allows a seizure to occur. Increased brain excitability is reflected by interictal discharges, seizures, and pathological network oscillations [61,62]. While an individual neuron might fire in an epileptic pattern (i.e., rapid, repetitive, paroxysmal discharges), a seizure is inherently a network phenomenon that entails numerous neurons firing simultaneously. Any brain region can potentially generate a seizure under certain conditions, that is, when net excitation in a cortical area exceeds net inhibition in that area. Each step in the sequence of seizure initiation, propagation, and termination is ultimately governed by the E/I balance in a specific neuronal circuitry. This is likely a manifestation of the summation of the dynamic and complex interaction between neurons, glia, vascular components, and the extracellular milieu.

In GE, defects caused by a gene mutation can directly or indirectly alter E/I balance, tilting the brain into a more excitable state. The identification of mutations in both non-ion and ion channel genes suggests the complexity of epilepsy, but this provides critical insights into understanding the generation of epileptiform activity. Many gene products involved in metabolism, which is fundamentally important for cell growth, function, and homeostasis, have recently been identified to regulate neuronal excitability, and their dysfunction is associated with GE. Mutations in *SLC2A1* encoding glucose transporter protein 1 (Glu1) are a good example [63]. Mutations in *Glu1* cause deficiency in glucose transport, thus a reduced energy supply for cells, which can be amenable with a diet using an alternate source of energy, such as in a ketogenic diet (KD) [64]. Not only do hyperexcitable or seizing brain regions require and utilize excess energy, but genetic or acquired metabolic alterations can also engender seizures, leading to the conclusion that metabolic dysfunction is both a cause and consequence of epilepsy [65]. This may help explain the increased brain excitability in many other neurological conditions, such as AD, which does not have a genetic mutation that directly alters ion flow and membrane excitability in the entailed neurons.

Normal brain functioning requires a fine E/I balance such that any subtle disturbance in this balance could tilt the brain to seizure or a seizure-prone state. We have demonstrated that a small alteration of GABA_A_ receptor expression can result in a dramatic difference at the behavioral level [30,60]. This notion is supported by the observation that exposures or pathologies that slightly increase excitation or reduce inhibition are ictogenic. Indeed, the changes of various GABA_A_ receptors have been observed in nongenetic-related human epilepsies [66,67,68]. This also explains why seizures are commonly observed in conditions including acute metabolic disturbances, such as hypoglycemia, stroke, or brain infections. However, the notion of the E/I imbalance may be challenged when considering chronic epilepsy and its numerous etiologies and diverse phenotypes. It is intriguing that patients with a permanently altered E/I imbalance only have seizures intermittently, not constantly. This merits further study to elucidate the complex and dynamic process that ensures a constant E/I homeostasis or how the delicate balance is disturbed in disease conditions such as epilepsy and AD.

## 8. Insights from Ketogenic Diet Treatment in GE into Understanding E/I Imbalance and Seizure Occurrence in AD

The identification of many non-ion channel gene mutations in GE and the occurrence of seizure activity in metabolic disorders suggest that dysregulated E/I balance could take place in various conditions without obvious disruption of ion flows at the cell membrane. It is not surprising that a diseased condition in AD entails E/I imbalance. Some insights can be gained from *SLC2A1* mutation-mediated seizures or a Glu1 deficiency that is responsive to KD [69]. The successful control of epilepsy with KD represents a proof-of-principle example of how metabolism alters neuronal excitability and seizure activity. Thus, the metabolic regulation of excitability and epilepsy must be taken into consideration for understanding E/I in AD. This may well explain the generation of epileptiform activity in dementia without obvious genetic risk factors. A KD diet, involving a high (4:1) ratio of dietary fat to carbohydrates (by weight), has been used for control of drug-refractory pediatric seizures. Efforts to unravel the mechanism(s) by which the KD prevents seizures have been ongoing. However, how a brain becomes hyperexcitable or epileptogenic is likely a complex process, much like the effect of KD in epilepsy. A simple E/I imbalance does not completely explain the KD’s mechanism of action. Rather, multiple molecular underpinnings are likely at play. These likely include key molecules involved in membrane excitability and energy metabolism, such as adenosine, ATP-sensitive K^+^ channels, and mitochondrial function. In GE, mutations in genes governing any of the above mechanisms can predispose the brain to a hyperexcitable state or a seizure-prone state. In neurodegenerative diseases—such as AD in the geriatric population with many years of disease-predisposing factors, including the accumulation of Aβ or oxidative stress—changes in metabolism over time may cause the dysfunction of neurons and a predisposition to seizure generation.

## 9. Insights from GE into Understanding Impaired Cognition in AD

In GE, many gene mutations are associated with refractory epilepsy and impaired cognition. This group of epilepsy is called epileptic encephalopathy and includes Dravet syndrome (DS), Lennox-Gastaut syndrome, infantile spasm, Ohatahara syndrome, etc., and is caused by mutations in a variety of genes [70,71,72,73]. It is not clear why some mutations can cause such a severe epilepsy phenotype while other mutations in the same genes can only cause a mild seizure phenotype, such as childhood absence epilepsy and febrile seizures. It is plausible that those gene mutations associated with more severe phenotypes may cause more damage to the biological function of the afflicted genes. Indeed, studies on *GABRB3* mutations suggest that the mutation that causes more impaired synaptic clustering of functional GABA_A_ receptors has a more severe phenotype than the mutation that causes a partial loss of function and less reduced synaptic clustering [74]. However, this correlation is mutant subunit dependent or even variant dependent for the mutations in the same gene. This notion is reinforced by the wide phenotypic spectrum among loss-of-function mutations, even in the same gene. For example, nonsense mutations in *GABRG2* associated with impaired cognition have mutant subunit protein accumulation and aggregation, while other nonsense mutations in *GABRG2* without mutant subunit protein accumulation and aggregation are associated with much milder epilepsy syndromes, such as childhood absence epilepsy or simple febrile seizures [73]. Regardless, a more severely impaired synaptic receptor distribution due to *GABR* mutations, regardless of whether in *GABRB3* or *GABRG2,* is correlated with impaired cognition, which is likely a final outcome or an overall summation of damage in the involved neuronal network. This is reminiscent of impaired synaptic physiology due to the altered synaptic structure or function in AD.

## 10. Endoplasmic Reticulum (ER) Stress Exists in Both GE and AD

The ER is important for the maintenance of intracellular Ca^2+^. The disruption of Ca^2+^ levels can critically affect mitochondrial function, glutamatergic AMPA and NMDA receptors, and synaptic signaling. ER stress in AD is thought to occur primarily following the accumulation of misfolded proteins, including Aβ, inducing a Ca^2+^ imbalance within the cell [75,76]. This dysfunction then impairs membrane-bound protein trafficking and disrupts the protein quality control machinery of the cell. Sustained ER stress will lead to failure of proteostasis and emergence of protein aggregation in neurodegenerative diseases such as AD. We have extensively studied the mouse models of GE with mutations in GABA_A_ receptor subunit genes, and we found that the *GABRG2(Q390X)* mutation, which led to the aggregation of GABA receptor γ2 subunit and increased ER stress, was associated with a more severe seizure phenotype [77]. Additionally, the *Gabrg2^+/Q390X^* mice had increased mortality and impaired cognition compared to the *Gabrg2^+/−^* mouse with a loss-of-function mutation in the same protein that did not cause ER stress [60,78]. Importantly, we identified increased neuroinflammation in the *Gabrg2^+/Q390X^* mice but not in the *Gabrg2^+/−^* mouse without ER stress [79]. The loss-of-function model has only mild epilepsy with normal cognition. In the same line, pharmacologically increasing ER stress resulted in memory deficits and impaired synaptic plasticity [80], suggesting a close link between ER stress and neuropathological processes, which are central to AD. 

## 11. ER Stress Leads to Impaired Membrane Protein Trafficking and Altered Synaptic Physiology

As mentioned above, it has been established that protein misfolding and aggregation is a common feature for neurodegenerative diseases. Metabolism of the misfolded protein, either resulting from gene mutation or other molecular errors, will engage the same intracellular protein quality control machinery. This is essential for protein folding, biogenesis, and trafficking. Our studies on *GABRG2* mutations indicate that ER stress reduces membrane protein trafficking and synaptic function [30]. In AD, the mutant protein inhibits protein folding, exacerbates the consequence of tau aggregation, and accelerates the misfolding and aggregation of other structurally challenged proteins with mutations, errors, and structural instabilities. In our studies, the *GABRG2(Q390X)* mutation associated with severe epilepsy diminished the expression of the wildtype partnering subunits and reduced the function of the remaining GABA_A_ receptors. When compared with the mice harboring a mutation causing ER stress or a mutation not causing ER stress, there are changes at multiple levels from protein synthesis to GABA_A_ receptor expression, and from channel function to synaptic GABAergic neurotransmission.

In the mutation that does not cause ER stress, the mice only manifested mild seizures and had normal cognition, while the mice with the mutation-causing ER stress manifested severe epilepsy and impaired cognition [60]. 

## 12. ER Stress, Proteostasis, and Reduced Membrane Protein Trafficking in AD

AD is progressive, and synaptic dysfunction and accumulation of abnormal aggregates formed by Aβ deposits or phosphorylated tau proteins are the major pathologies. Mounting evidence suggests that increased ER stress and alterations in the buffering capacity of the proteostasis network are a salient feature of AD. The ER is the main compartment involved in protein folding and is drastically affected in AD. ER stress triggers the activation of the unfolded protein response (UPR), a signal transduction pathway that enforces adaptive programs to recover homeostasis or trigger apoptosis. In addition to Aβ plaque formation and deposition, tauopathy and/or increased phosphorylated tau is another signature of dementia. The microtubule-associated protein tau in its abnormally hyperphosphorylated form is the major protein subunit of the paired helical filaments, which are glycosylated in AD brain tissues but not in normal controls [81]. It is unclear how increased tau aggregation and ER stress affect membrane protein trafficking in AD. A recent study indicates that Tau aggregates inhibit the protein-folding and vesicular trafficking arm of the cellular proteostasis network [82]. In our studies on *GABRG2* loss-of-function epilepsy mutations with or without ER stress, a correlation of epilepsy phenotype severity with ER stress has been identified [60,77].

In AD cell models, excessive amounts of misfolded or unfolded proteins lead to an activation of the UPR, and there is a close link between ER stress and bioenergetics defects under normal conditions [83]. Additionally, studies from induced pluripotent stem cell (iPSC) have provided new insights. For example, iPSC-derived neurons carrying PERK risk alleles (protein kinase R-like ER kinase) were highly vulnerable to ER stress-induced injury with increased tau pathology. Along the same line, the chemical inhibition of PERK in human iPSC-derived neurons also increased neuronal cell death in response to ER stress. Down syndrome individuals have an increased risk of developing AD-like pathology and dementia by the age of 40 due to the triplication of several genes involved in the formation of amyloid plaques and tau tangles [84,85]. It has been established that the accumulation of misfolded proteins in the ER triggers UPR. Long-term activation of the UPR mediates neuronal dysfunction, and sustained ER stress can cause neuronal death in AD. In a mouse model, the activation of the PERK pathway in Ts65Dn DS mice was identified at a much younger age than in the normal controls, suggesting that UPR activation at an early phase of disease might be an essential contributor to the failure of proteostasis. In a *Gabrg2^+/Q390X^* knockin mouse model of epilepsy, we identified mutant protein accumulation in newborn mouse pups, but the mutant protein aggregates did not emerge until later in life [86]. This thus suggests that ER stress could exist many years before disease onset as the emergence of the clinical symptom of cognitive deficit. In summary, ER stress is likely an early event in AD, while persistent ER stress leads to neuronal loss.

## 13. Increased ER Stress Can Cause Increased Neuroinflammation from Very Early on: Evidence from an Epilepsy Mouse *Gabrg2^+/Q390X^*

We have extensively characterized mutant protein metabolism in GE caused by gene mutations affecting the GABAergic pathway, primarily in GABA_A_ receptor and transporter 1 genes. It is common that mutations in both GABA receptors and transporters result in mutant protein that is retained inside ER, causes ER stress, and exacerbates epilepsy phenotype [77]. In *Gabrg2^+/Q390X^* epilepsy mouse models, the existence of the mutant protein that is aggregation prone with slow degradation promotes a mild infrequent absence to a much severe epilepsy phenotype of DS with increased seizure severity and cognitive impairment [60]. We recently identified that the mutant protein caused increased ER stress in the *Gabrg2^+/Q390X^* mouse and caused increased neuroinflammation, as evidenced by inflammatory cytokines such as tumor necrosis factor (TNF) alpha, interleukin 1-beta, and interleukin 6 in the newborn pups [79]. Given that neuroinflammation is an established patho-mechanism of AD [87,88] and TBI [89], this thus provides a direct link between GE and AD as well as TBI, a risk factor for both epilepsy and AD. It is likely that multiple etiologies can cause ER stress and increase neuroinflammation. This also suggests a unique opportunity to target ER stress and reverse disease pathology.

## 14. Impaired GABAergic Signaling Is a Converging Pathway of Pathophysiology in GE

GABAergic signaling is an established pathway for seizure generation for both genetic and acquired epilepsies. Thus, it is not surprising that GABAergic neurotransmission is involved in the pathogenesis of AD. In the pediatric population, many mutations in both ion channel and non-ion channel genes have been identified as impairing GABAergic signaling. These mutations can directly or indirectly affect GABAergic neurotransmission. The mechanisms by which these genetic mutations impair GABAergic signaling include pre- and postsynaptic mechanisms. The epilepsy genes that impair GABAergic signaling via the presynaptic mechanisms include, but are not limited to, *STXBP1*, *STX1B*, *DNM1,* and *PRRT2* [90]. These genes encode proteins that are involved in vesicle fusion machinery and vesicle release. The defects in the vesicle fusion machinery affect the presynaptic vesicle release. Failure or impaired release of key neurotransmitters would profoundly impair the corresponding neurotransmission and synaptic activity. In contrast to genetic epilepsy, impairment directly caused by mutation is the causing factor for the afflicted pediatric population. In AD, there may be acquired factors, such as altered metabolism, neuroinflammation, and Aβ deposits, that affect similar components in the GABAergic pathway instead of a genetic mutation. 

## 15. Altered GABAergic Neurotransmission Including GABA Neurotransmitter, Receptors, and Transporters in AD

**Figure 1:**
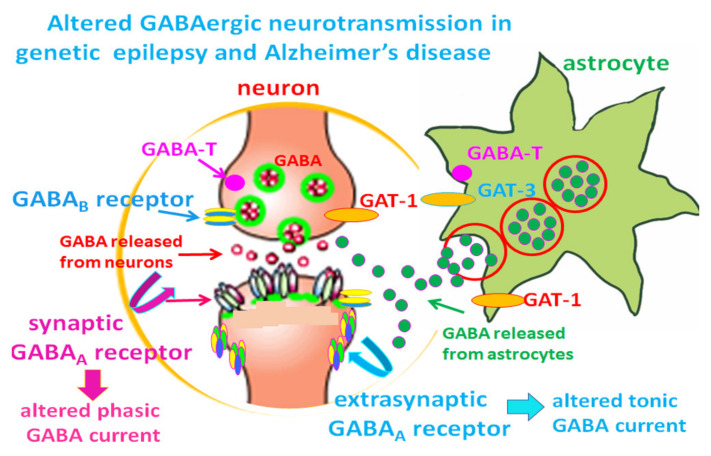
Altered GABAergic neurotransmission in both genetic epilepsy and Alzheimer’s disease. The neurotransmitter GABA can be released from both neurons and glia. GABA is synthesized from glutamic acid, the principal excitatory neurotransmitter via glutamic acid decarboxylase (GAD). GABA is catabolized by GABA transaminase (GABA-T), which is a membrane-bound enzyme expressed by neurons and glia. In GABAergic interneurons, GABA is released from vesicles in presynaptic terminals and activates GABA receptors, which include GABA_A_ receptors and GABA_B_ receptors. Under pathological conditions, such as AD, GABA can also be released from reactive astrocytes. GABA_A_ receptors hyperpolarize neurons via Cl^−^ influx. The released GABA is taken up by GABA transporters (GAT-1 and GAT-3) back into presynaptic compartments of neurons or into astrocytes. Failure of GABA clearance due to malfunctioning GABA transporters or excessive GABA production by reactive astrocytes will alter GABAergic neurotransmission, leading to a seizure-prone brain state [90].

GABA is the major inhibitory neurotransmitter, while glutamate is the major excitatory neurotransmitter in the brain. Both neurotransmitters work together to control many neuronal processes, including the overall brain excitation and synaptic homeostasis. It has been established that glutamic acid decarboxylase (GAD) converts glutamate to GABA. There are two isoforms of GAD—GAD65 and GAD67—that synthesize GABA in the brain. After being released from presynaptic terminals, GABA is taken up by GABA transporters 1–4. These transporters are widely expressed in neuronal (mainly GAT-1) and glial (mainly GAT-3) cells throughout the brain, including the hippocampus [91]. Inside the cell, GABA is degraded by GABA transaminase to succinic semialdehyde, and inhibition of this enzyme by antiseizure drugs (ASD), such as vigabatrin, increases GABAergic neurotransmission by increasing the GABA level. An impaired GABAergic pathway is a major pathology in genetic epilepsy. Understanding this pathway from genetic epilepsy could provide insights into the pathophysiology of AD (Figure 1).

## 16. Impaired GABAergic Interneurons in AD

GABAergic neurotransmission includes the presynaptic release of GABA and activation of the GABA_A_ receptor via postsynaptic mechanisms. GABA is released by GABAergic interneurons that provide much of the inhibition in multiple brain regions, such as the cerebral cortex, hippocampus, striatum, and amygdala. Impaired interneuron function has been established as an underlying cause for epilepsy in multiple preclinical animal models [92]. Along the same line, much effort has been taken to rescue interneuron function for treating epilepsy. For example, it has been reported that GABA progenitor cells grafted onto the adult epileptic brain attenuated seizures and comorbidities in mice [93,94]. In AD, the mutation-induced loss of APP function causes GABAergic depletion in the recessive familial disease in a knockin mouse carrying the E693Δ (Osaka) mutation [6]. The mutant homozygote mice displayed the intraneuronal accumulation of Aβ oligomers at 8 months, followed by abnormal tau phosphorylation, synapse loss, glial activation, and neuron loss. The levels of GABA-related proteins and the number of dentate GABAergic interneurons were decreased in 4-month-old homozygotes. Overall, the findings from this mouse model indicate a GABAergic deficit in AD with the Osaka mutation. A recent study also has reported that only a subtle dysfunction of parvalbumin interneurons was identified in an *APPN^L-F^* knockin mouse model of Alzheimer’s disease [95]. Along the same line, Nav1.1-overexpressing interneuron transplants restore brain rhythms and cognition in a mouse model of Alzheimer’s Disease. An increased Nav1.1 level accelerated the action potential kinetics of transplanted fast-spiking and non-fast-spiking interneurons [96]. This is not surprising given the biological role of sodium channel Scn1a in GABAergic interneurons.

## 17. Altered GABA Level and Tonic Inhibition in AD

It is worth noting that it has been identified that GABA content is high in reactive astrocytes [97]. Synaptic inhibition is based on both tonic and phasic release of the neurotransmitter GABA. Although phasic GABA release arises from Ca(2+)-dependent exocytosis from neurons, the mechanism of tonic GABA release is not totally clear. It has been found that in both human and mouse AD brains, there are increased GABA levels [97] and increased tonic inhibition [98]. Functional and behavioral analysis demonstrated that increased tonic inhibition suppresses long-term potentiation and results in memory deficit in 5XFAD mice. The increased GABA is due to increased GABA release from glial cells by permeation through the Bestrophin 1 (Best1) anion channel [99] or by GAT-3/4, which has been demonstrated to be reversed to release GABA in astrocytes upon stimulation of glutamate [100]. It has been demonstrated that GABA directly permeates through Best1 to yield GABA release and that tonic inhibition is eliminated by the silencing of Best1. Glial cells express both GABA and Best1, and the selective expression of Best1 in glial cells, after preventing the general expression of Best1, fully rescues tonic inhibition [99]. The data suggest close interactions between glia and neurons in mediating tonic inhibition and seizure generation. The finding of an increased GABA level in AD merits further study, which may benefit from tools and animal models developed from genetic epilepsy with defective GABA transporters, such as those caused by *SLC6A1* mutations [51,52]. This will help tease out the contribution from the malfunctioning GABA transporter or GABA receptors by modulating the expression of the GABA transporter or receptors.

The hallmark pathology of AD is Aβ-deposition and tau pathology that are thought to ultimately lead to synapse and neuron loss. Despite their possible causative role, the consensus is that targeting amyloidosis, tau phosphorylation, acetylcholine esterase, glutamate, oxidative stress, and mitochondrial metabolism has not yet led to the development of drugs to cure AD. Recent preclinical and clinical reports exhibit a surge in interest in the role of GABAergic neurotransmission in the pathogenesis of AD. The interaction among GABAergic signaling, Aβ, and acetylcholine is shown to affect the homeostasis between excitation (glutamate) and inhibition (GABA) in the brain. Previously, the glutamate arm of this balance received the most attention, and it is likely that overexcitation due to excitoxicity that leads to neurodegeneration and subsequent neuronal loss contributes to the cognition deficit. Recent studies suggest that overexcitation is primarily mediated by altered GABA signaling and can possibly be restored by modifying the function involved in the GABAergic neurons during AD [6]. Additionally, it has demonstrated that GABAergic signaling can affect neurogenesis and synaptogenesis [101]. Furthermore, several preclinical interventional studies revealed that pharmacological approaches that target various GABA receptor subtypes hold promise for treatment against the memory deficits associated with AD [102].

## 18. Treatment Opportunities for AD

**Figure 2:**
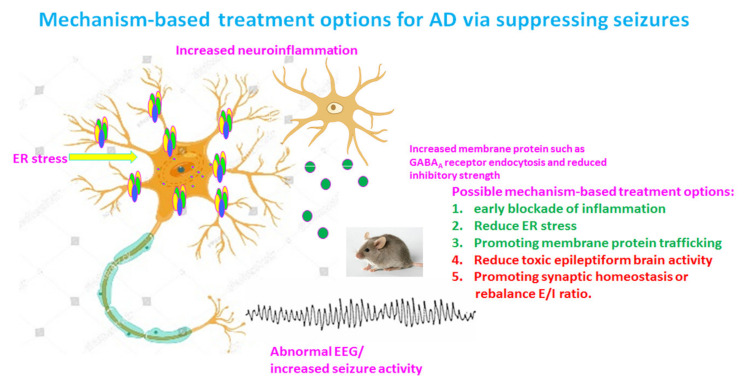
Potential mechanism-based treatment options for Alzheimer’s disease (AD) via suppressing toxic epileptiform discharges. There are multiple pathologies in a chronic disease state such as AD. In addition to the established amyloid hypothesis and tauopathy, pathological features for AD likely also include increased neuroinflammation, endoplasmic reticulum (ER) stress, impaired protein membrane trafficking, and increased toxic epileptiform discharges due to altered brain excitation and inhibition (E/I) balance. Therefore, any pharmacological or genetic approaches that can block neuroinflammation, reduce ER stress, promote vesicular protein trafficking, or correct E/I imbalance would be beneficial and disease modifying for AD.

The landscape of AD therapeutics remains bleak despite enormous efforts. Until recently, the only available drugs for this condition were cholinergic treatments, which symptomatically enhance cognitive state to some degree, but these compounds were not neuroprotective nor disease modifying. Many other treatment options have been proposed via a diversity of mechanisms and reflect the multifactorial pathophysiology of AD well (Figure 2). These treatment options include reagents against antioxidative stress, neuroinflammation, and excitotology, etc.

## 19. Targeting GABAergic Neurotransmission as Treatment for AD

A recent study suggests that GABAergic signaling presents itself as a promising target for treatment development for AD in addition to other drug targets. Impairment of the GABAergic system is essentially involved in the pathogenesis of AD from early-phase neuroinflammation to the emergence of late-phase cognitive deficit. Traditionally, agonists of GABA_A_ receptors at doses above 1 mg/kg are known to possess memory-impairing effects. However, it has been demonstrated that in an AD model of rats, the GABA_A_ receptor GABA binding site ligand muscimol enhanced spatial learning/memory at very low doses. An α5 subunit containing a GABA_A_ receptor antagonist showed a persistent therapeutic effect in a rodent preclinical model of vascular cognitive impairment [103]. A study also found that the effects of nonsedative—very low (0.05 mg/kg) and moderate (1 mg/kg)—doses of diazepam, a positive allosteric modulator of GABA_A_ receptors, are protective for memory in a rat AD model. Consistently, the GABA-containing compound gammapyrone protects against brain impairments in an AD cultured model [104]. In nontransgenic AD model rats (intracerebroventricular streptozocin injection), a model aiming to mimic the predementia stage of AD in humans, diazepam protected against streptozocin-induced symptoms by enhancing spatial learning/memory, reducing neuroinflammation, and preserving synaptic plasticity, and the treatment normalized the hippocampal and cortical protein expression related to acetylcholine breakdown and GABA biosynthesis. It is possible that at low and moderate doses, diazepam targets nonspecific, probably allosteric, GABA_A_ receptor sites, thereby leading to stimulatory effects that can be beneficial for diazepam use in early predementia stages of AD.

GABAergic neurons are known to increase neuronal inhibition and inhibit neural transduction and therefore negatively affect excitatory neural circuits in the brain. A previous report reported that 5-(3-methoxyphenyl)-3-(5-methyl-1,2,4-oxadiazol-3-yl)-1,6-naphthyridin-2(1H)-one (AC-3933), a partial inverse agonist for the benzodiazepine receptor, reverses the GABAergic inhibitory effect on cholinergic neurons and thus enhances acetylcholine release from these neurons in rat hippocampal slices. Oral administration of AC-3933 (0.01–0.03 mg/kg) resulted in the amelioration of scopolamine-induced or MK-801-induced amnesia [105], as well as a shift in the EEG relative power characteristic of pro-cognitive cholinergic activators, such as donepezil. In addition, treatment with AC-3933, even at the high dose of 100 mg/kg p.o., produced no major side effects such as seizure or anxiety. AC-3933 has thus been proposed to be used for AD treatment [106,107]. In preclinical mouse models, blocking GABA transporters with SNAP-5114 or L-655708 has been reported to improve memory in AD mice [98].

## 20. Targeting ER Stress as a Treatment Option in AD

As aforementioned, AD is characterized by severe cognitive impairment and memory loss. At least in some AD patients, ER stress may exist long before the emergence of cognition impairment. At the molecular level, AD is classified both into the “protein conformational” and the “ER-mitochondria stress” disorders. From the pathophysiology point of view, the amyloid hypothesis of AD cannot fully explain the diverse clinical forms of the disease, and the failure of Aβ clearance seems to reinforce this notion. Protein folding and misfolding in the ER and the accumulation of several misfolded proteins, such as Aβ, Tau, alpha-synuclein, etc., in the ER and mitochondria may play a key role in the development of AD. Mitochondria and ER are tightly coupled both physically and functionally with a special lipid raft called the mitochondria-associated ER membrane, which is crucial for Ca(2+) signaling and the metabolic regulation of the cell. In turn, the impairment of ER–mitochondria interplay is a common mechanism of different neurodegenerative diseases. Although there is no treatment to directly target ER stress, studies have demonstrated that enhancing ER stress can directly impair memory and synaptic plasticity [80]. Although there is no direct evidence, it is plausible that compounds including chemical chaperones, such as 4-phenylbutyrate acid, that relieve ER stress via enhancing refolding may improve memory by reversing the pathophysiology caused by ER stress in AD.

## 21. Targeting Tau as a Treatment Option for AD and Insights from GE

It has been demonstrated that the deletion of tau reduced neuronal network hyperexcitability in mouse and Drosophila models of AD [108]. It is intriguing that the interaction between tau and network excitability is robust in AD animal models with Aβ or tau pathology. It is not clear why tau can modulate neuronal excitability in models without aberrant amyloid expression or tau hyperphosphorylation. In animals without Aβ or tau pathology, tau knockout also showed a decrease of seizure severity following a challenge with convulsants [22,109]. If tau removal plays a more general role in reducing neuronal network firing, targeting tau could be a viable target for AD as well as for other hyperexcitability conditions. This notion was supported by the deletion tau in kcna1^−/−^ mice. The mice exhibited severe spontaneous seizures starting from the third week of life. The mice had early lethality and megencephaly, whereas the reduction of tau increased survival, prevented megencephaly, and reduced hyperexcitability [108]. Consistently, a study on a *Scn1a^+/R1407X^* knockin mouse model of Dravet syndrome (DS), a severe developmental epileptic encephalopathy, demonstrated that the deletion of tau rescued the disease phenotype. Similarly, tau ablation in Scna1^−/−^ mice prevented the high mortality of DS and reduced the frequency of spontaneous and febrile seizures [110]. It reduced interictal epileptic spikes in vivo and drug-induced epileptic activity in brain slices ex vivo. Additionally, tau ablation prevented biochemical changes in the hippocampus, indicative of epileptic activity and improved learning and memory, nest building, and open field behaviors, in DS mice. The deletion of only one copy of a tau allele was sufficient to attenuate disease symptoms [110]. The findings from GE and AD in combination thus suggest that tau reduction could be a viable target for AD and at least some GEs.

## 22. Targeting Glutamatergic Neurotransmission as a Treatment Option for AD

It is logical to test neuroprotective drugs for AD, but many trials have failed due to no efficacy or severe side effects. However, one putatively neuroprotective drug, the adamantane derivative memantine, showed promise for the treatment of dementia. Recent phase 3 clinical trials have shown that memantine is effective in the treatment of both mild and moderate-to-severe AD and possibly vascular dementia. This suggests its potential broad application in cognitive dysfunction. Memantine has also been used in GE and intellectual disabilities associated with mutations affecting N-methyl-D-aspartate (NMDA)-type glutamate receptors, such as mutations in GRIN2A [111]. It has been reported that the transfection of cultured neurons with GluN2A-P552R prolonged EPSPs and triggered pronounced dendritic swelling in addition to excitotoxicity, which were both attenuated by memantine [112]. The primary mechanism of action of memantine is to block excitotoxicity. The overactivation of NMDA receptors results in excessive Ca(2+) influx through the receptor-associated ion channel and subsequent free radical formation, leading to neuronal injury or neuronal death. Memantine can preferentially block excessive NMDA receptor activity without compromising normal neuronal activity. It is encouraging that memantine is well tolerated in AD patients [113,114], suggesting its broad clinical application. Due to the efficacy and tolerability of memantine in clinical application, a series of second-generation memantine derivatives is currently in development in hope of achieving better efficacy and tolerability [115]. In addition to NMDA receptors that can be targeted by memantine, the kynurenine pathway (KP) has been implicated in the development of neurodegenerative processes and is reported to be altered in both acute and chronic neurological disorders. The neuroprotective metabolite, kynurenic acid, has been associated with antagonistic effects at glutamate receptors. Additionally, it can also exert neuroprotection via its free radical scavenging and immunomodulation [116]. Thus, the KP offers another therapeutic target, at least partially through the attenuation of glutamatergic excitotoxicity and neuroprotection.

## 23. Conclusions

AD pathophysiology involves a multifactorial process, which includes Aβ deposits, tau aggregates, excitotoxicity, and neuroinflammation, etc. Despite its late-onset disease course, AD may have significant overlaps with GE regarding the neural mechanisms and signaling cascades governing membrane protein trafficking (Table 1). Advances from GE may provide unique opportunities for understanding AD. In GE, a specific mutation from conception and the early onset of disease can provide a much cleaner picture of the involved pathway that is obscured in AD over a chronic disease course. Understanding the biological role of these gene mutations and findings from corresponding knockin mouse models would provide novel critical insights into AD pathophysiology and therapeutic developments.

Some insights into seizure manifestation and protein aggregation can be gained from the *Gabrg2^+/Q390X^* knockin mice of GE. The study on *Gabrg2^+/Q390X^* mice implicates that mutant protein accumulation may exist from the beginning of life, although the protein aggregates will not emerge until later in life [30]. Importantly, increased neuroinflammation due to ER-retained mutant protein was observed in knockin mouse pups carrying the GABRG2(Q390X) mutation, suggesting that underlying changes occur long before any symptom emerges [79]. The efficacy of memantine in epilepsy and cognitive improvement with *GRIN2A* mutations does a good job of explaining why the blockade of NMDA receptors is beneficial for AD. The mechanism of action of memantine is likely to block NMDA channel function, which consequently suppresses epileptiform discharges and protects against memory loss. The success of the pharmacological application in AD in return may help tailor the treatment or symptom management for GE. Furthermore, the discovery of multiple epilepsy genes with diverse biological functions in GE helps explain the complexity of AD pathophysiology. This suggests that many factors and signaling pathways could converge and contribute to the same altered E/I balance. An altered E/I balance can tilt the brain to hyperexcitability, leading to seizures and cognitive deficit in AD. Thus, this will open more avenues for developing treatment options for AD.

## Figures and Tables

**Table 1:** Epileptic mechanisms in Alzheimer’s disease.

Gene	Mutation/Variants	Models	Postulated Mechanisms	Channel Function	Phenotypes	References
*GABRG2*	Q390X	cells	Impaired oligomerization, ER retention	reduced	GEFS+, DS	[30]
*GABRG2*	R82Q	cells	Impaired oligomerization, ER Retention	reduced	FS, CAE	[117]
*GABRG2*	Q390X, W429X, W461X	cells	Impaired oligomerization, ER Retention	reduced	FS, GEFS+, DS	[77]
*GABRG2*	Q390X	mice	ER rentention, dominant negative suppression	reduced		[62]
*GABRG2*	Q390X	Mice	protein accumulation, aggregation	reduced	GEFS+, DS	[118]
*GABRG2*	Q390X	mice	increased neuroinflamamtion	reduced	DS	[79]
*GABRG2*	IVS6+2T->G	cell mice	NMD, ERAD	reduced	CAE, FS	[119]
*GABRG2*	A106T, I107T, P282S, etc.	cell	ERAD, ER retention	reduced	DEE	[120]
*GABRG2*	S443delC	cells	ERAD, ER retention?	reduced	GEFS+	[121]
*GABRB3*	N328D	cells	ERAD, ER retention	reduced	LGS	[74]
*GABRB3*	E357K	cells	ERAD, ER retention	reduced	JAE	[74]
*GABRA1*	A322D	cells	ERAD, ER retention	reduced	JME	[122]
*SLC6A1*	G234S	cells	ERAD, ER retention	reduced	DS	[51]
*SLC6A1*	P361T	cells	ERAD, ER retention	reduced	autism, CAE	[52]
*SLC6A1*	V125M	cells	ERAD, ER Retention	reduced	CAE, ADHD	[123]
*SLC6A1*	22 mutations	cells	ER Retention, dominant negative effect?	reduced	various phenotypes	[53]

Abbreviations: CAE = childhood absence epilepsy; FS = febrile seizures; GEFS+ = generalized epilepsy with febrile seizures plus; DEE = developmental epileptic encephalopathy; LGS = Lennox-Gastaut syndrome, DS = Dravet syndrome; JAE = juvenile absence epilepsy; ADHD = attention deficit hyperactivity disorder.

## Data Availability

Not applicable.
